# COVID-19 Infection Or Buttock Injections? the Dangers of Aesthetics and Socializing During a Pandemic

**DOI:** 10.2478/jccm-2021-0043

**Published:** 2022-02-09

**Authors:** Derrick Anthony Cleland, Clarence H. H. Tsai, Joslyn Vo, Dafne Moretta

**Affiliations:** 1 Loma Linda University Medical Center Loma Linda, CA, USA; 2 Loma Linda University School of Medicine, Loma Linda, CA, USA

**Keywords:** silicone embolism syndrome, acute respiratory distress syndrome, diffuse alveolar hemorrhage, critical care medicine, COVID-19

## Abstract

**Introduction:**

Silicone (polydimethylsiloxane) injections are used for cosmetic augmentation. Their use is associated with life-threatening complications such as acute pneumonitis, alveolar hemorrhage, and acute respiratory distress among others [1,2]. We report a case of a Hispanic woman who developed severe respiratory distress syndrome after gluteal silicone injections.

**Case Presentation:**

A 44-year-old Hispanic female presented to the Emergency Department complaining of progressive dyspnea on exertion for two weeks. Chest imaging revealed patchy bibasilar airspace opacities of peripheral distribution. Labs were significant for leukocytosis, elevated PT, D-dimer, lactate dehydrogenase, and fibrinogen, concerning for COVID-19, however SARS-CoV-2 testing was negative multiple times. The patient later became encephalopathic, hypoxemic, and eventually required intubation. Further history uncovered that the patient had received illicit gluteal silicone injections a few days prior to her onset of symptoms. The patient was diagnosed with silicone embolism syndrome (SES) and initiated on high dose intravenous methylprednisolone [1].

**Case Discussion:**

Patients from lower socioeconomic backgrounds utilize illicit services to receive silicone injections at minimal costs. This leads to dangerous outcomes. The serology and imaging findings observed in our case have similarities to the typical presentation of COVID-19 pneumonia making the initial diagnosis difficult. This case serves as a cautionary tale of the importance of thorough history taking in patients with concern for COVID-19.

## Introduction

The ISAPS (International Society of Aesthetic Plastic Surgery) 2019 report demonstrated that there was a 10.4 % global increase in noninvasive aesthetic treatments, such as fillers, from the previous year. Silicone (polydimethylsiloxane) injections were originally thought to be ideal for cosmetic augmentation given its durability, stability, and lack of immunogenicity. Therefore, it has been used for cosmetic procedures by both medical and nonmedical personnel. However, the use of these treatments has been associated with several life-threatening complications such as acute pneumonitis, alveolar hemorrhage, and acute respiratory distress, among others [1,2].

Respiratory symptoms are the most predominant and usually manifest within several days from initial exposure but reactions have been seen up to a year following injection [3,4]. The pathophysiological mechanism is thought to be due to an inflammatory response and cellular damage in response to systemic spread of silicone emboli [5]. Treatment is supportive but often requires a steroid regimen [4]. The use of clandestine silicone injections therefore, creates potential risks for susceptible populations.

## Case Presentation

**Fig. 1:**
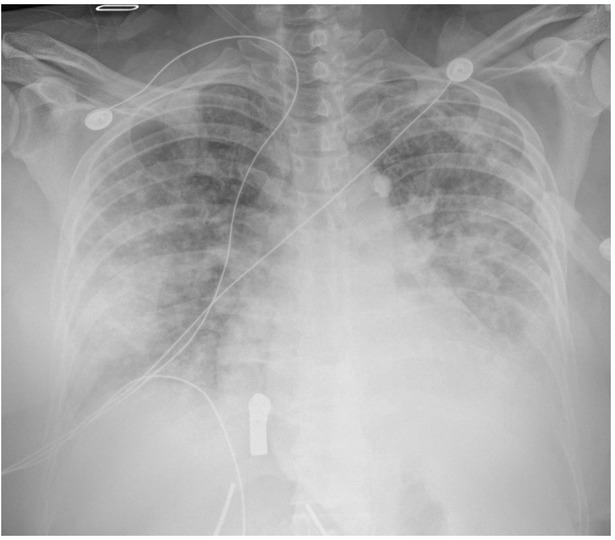
Chest X-Ray (Single View) on Day 3 of Hospitalization

**Fig. 2:**
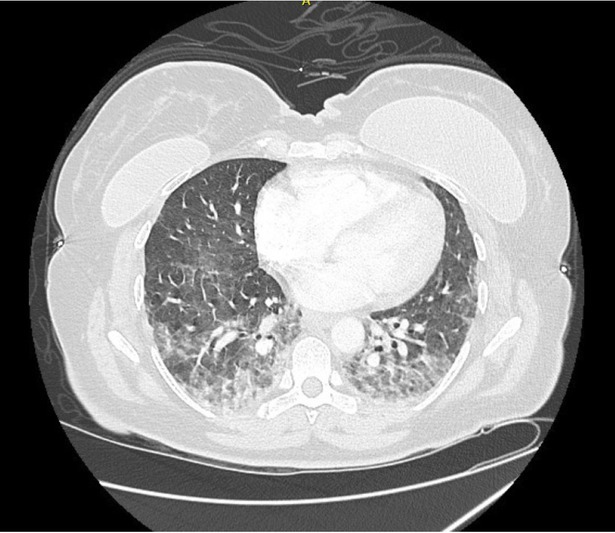
Chest CT Angiography on Day 2 Of Hospitalization

A 44-year-old Hispanic female with a history of anxiety presented to the Emergency Department with a two-week history of exertional dyspnea. She also had associated dry cough, chills, pleurisy, lightheadedness, and fatigue. At the time of presentation to the emergency department, her vitals were notable for tachypnea and hypoxia, requiring a high-flow nasal cannula. In addition, a chest x-ray revealed patchy bibasilar airspace opacities with peripheral involvement (see appendix, figure 1). A CT pulmonary angiography (CTPA) revealed bibasilar ground glass airspace disease without evidence of filling defects (see appendix, figure 2). Laboratory tests on arrival were notable for leukocytosis, elevated prothrombin time (PT), D-dimer, lactate dehydrogenase (LD), and fibrinogen (see appendix, table 1).

On day of admission to the general wards, the patient’s presentation was concerning for COVID-19 and/or community-associated pneumonia, therefore, she was started empirically on Dexamethasone (6 mg, intravenously, daily [Mylan Laboratories Ltd., Hyderabad, IN], Low Molecular Weight Heparin (LMWH; enoxaparin; 40 mg, subcutaneously, daily [Meitheal Pharmaceuticals Inc., Chicago, USA]), Ceftriaxone (1 g, intravenously, daily [Sandoz GmbH, Kundl, AUT]) and Doxycycline hyclate (100 mg, oral, twice daily [Amneal Pharmaceuticals Pvt. Ltd., Ahmedabad, IND]). However, point of care (POC) and multiple SARS-CoV2 RNA tests returned negative on day 2 of admission.

On day 2 of admission, the patient became increasingly encephalopathic and hypoxic, and was subsequently intubated, placed on high ventilator settings, and transferred to the ICU for higher level of care (see appendix, table 2). Her hypoxia improved minimally with inhaled nitrous oxide (iNO) and proning on day 3 of admission. On day 4 of admission, she was found to have tracheal bloody secretions, therefore, bronchoscopy with bronchoalveolar lavage (BAL) was performed on day 5 of admission and revealed moderate erythema with residual blood seen throughout. Sequential BALs were consistent with diffuse alveolar hemorrhage with negative cultures (see appendix, table 3). An extensive autoimmune and infectious work-up was also performed on day 5 of admission and was unremarkable (see appendix, table(s) 4 & 5).

Further history was obtained from the patient’s family members on day 5 of admission, who stated that the patient had received bilateral gluteal and lip injections with silicone from an unlicensed professional. She had received these routinely over several years and a few days prior to her onset of symptoms. On receiving this information, it was concluded that the patient was experiencing diffuse alveolar hemorrhage secondary to suspected silicone embolism syndrome.

Following review of the literature on day 6 of admission, the patient was initiated on high-dose methylprednisolone (60 mg, intravenously, every 6 hours [Pharmacia & Upjohn Co, Division of Pfizer Inc., Kalamazoo, USA]) for management [1]. Initially, a lung biopsy was considered to confirm the diagnosis of silicone embolism syndrome. However, due to the patient’s high oxygen requirements, this was not carried out due to safety concerns and was postponed.

After ten days of high-dose steroids, from day 6 to day 15 of admission, the patient’s clinical status improved, and therefore, biopsy was deferred knowing that it would not change the overall outcome. The patient was eventually discharged home on a prednisone taper (Deltasone®; 40 mg, orally, daily for 7 days and then subsequent halving of dosage over the next 3 weeks [Oculus Innovative Sciences, Petaluma, USA]).

## Discussion

Over the past two decades, social media has increasingly glorified body image through the use of filters and Photoshop that alter the perceived physical appearance, resulting in more and more people turning toward augmentation as a way to achieve the ‘ideal’ body type. Buttock augmentation, in particular, has become very popular among women. Coincidentally, the number of legal buttock augmentation procedures in the United States has gone up 90% since 2015 [6].

However, the cost of these procedures ranges from several thousands of dollars (from $4,459 to $5,352) making it difficult for individuals from lower socioeconomic status to afford them [7]. Therefore, many turn to illegal plastic surgery clinics which perform the same procedures at a fraction of the cost. As a result, these patients put themselves at serious risk of health complications that may lead to life-threatening outcomes as has been reported by the media over the past several years [8]. Many of these illegal clinics do not use the proper equipment for administering these injections (eg, ultrasound guidance) which can lead to accidental puncture of a gluteal vessel or increased perivascular pressure leading to the development of silicone emboli [9]. Past studies have discovered that anti-silicone IgG antibodies can form immune complexes within the vasculature leading to an amplified inflammatory response [10]. As silicone invades the vasculature and forms immune complexes, these complexes can cause intravascular damage which can ultimately lead to the activation of the coagulation cascade.

The coagulopathy and elevated inflammatory markers observed in our case led to confusion early on when attempting to make a diagnosis because initial laboratory findings suggested a severe COVID-19 infection [11]. Additionally, the radiographic findings (eg, CTPA and CXR) of our patient were similar to those found in COVID-19 in which there is bilateral airspace disease with tendency to peripheral distribution [12]. The culmination of these findings made it difficult to distinguish between COVID-19 pneumonia versus SES. Therefore, it is important for physicians, regardless of the underlying cause, to utilize anticoagulation therapy when either SES or COVID-19 is suspected, as the use of LMWH can prevent the progression to disseminated intravascular coagulation (DIC); a lethal thrombotic event that may occur in either COVID-19 or SES [6]. High dose steroids should also be considered to reduce the inflammation that results from SES. Initially, we utilized the COVID-19 protocol steroid regimen (eg, Dexamethasone 6 mg, oral, daily), but did not see clinical improvement until high dose steroids were initiated, suggesting the need for a more aggressive regimen when treating SES.

## Conclusion

With the COVID-19 pandemic fresh in the mind of most physicians, recency bias played a role early in the clinical course of this patient, leading to increased costs and a delay in optimized treatment. Therefore, it is of the utmost importance for clinicians to obtain a thorough history when attempting to diagnose the cause of a patient’s respiratory failure so that the proper treatment measures can be initiated as soon as possible.

## Appendix

**Table 1:** Example of Inflammatory Markers Trended Early in Patient’s Hospital Course

	Day 1	Day 2	Day 3	Day 4	Day 5	Day 6
PT	12.8	N/A	15.3	N/A	15.0	N/A
D-dimer	1.6	1.3	1.6	1.3	4.4	> 21
LD	202	202	392	500	691	1,175
Fibrinogen	492	555	891	827	N/A	570

Abbreviations: PT=Prothrombin Time; LD= Lactate Dehydrogenase

**Table 2:** Ventilation Settings and Corresponding Arterial Blood Gas (ABG) Values

Hospital Day #	Ventilation Mode	PEEP (cm H2O)	Respiratory Rate (bpm)	FiO2 (%)	Tidal Volume (Vt; mL)	Arterial Blood Gas Values (pH/ paCO2/paO2/HCO3)	Ventilator Changes^2^
3^1^	AC/VC	12	16	100	400	7.33/39/184/19.7	↓ FiO2 to 50%
4	AC/VC	12	20	60	400	7.28/46/86/21.5	↓ FiO2 to 50%; ↑ RR to 26 bpm
5	AC/VC	16	26	100	390	7.32/43/133/21.7	↓ FiO2 to 90%; ↑ Vt to 400 mL
6	AC/VC	18	23	90	400	7.37/41/118/23.2	↓ FiO2 to 80%; ↑ Vt to 300 mL
7^3^	AC/VC	16	28	65	300	7.07/99/162/27.6	↓ FiO2 to 60%; ↑ RR to 32 bpm; ↑ Vt to 320 mL
8	AC/VC	10	38	70	320	7.30/62/117/29.6	↓ FiO2 to 60%
9	AC/VC	12	38	50	320	7.36/65/68/35.1	None
10	AC/VC	10	38	45	320	7.43/61/91/39.1	None
11	AC/VC	6	32	40	350	7.46/49/58/35.1	↑ FiO2 to 60%; ↑ PEEP to 8 cmH2O
12	AC/VC	8	32	45	350	7.44/48/110/32.0	↓ FiO2 to 40%; ↓ PEEP to 6 cmH2O
13	PS	6	N/A	30	N/A	7.46/42/71/29.0	Extubated successfully to NC, 6 LPM

Abbreviations: paCO2 = partial pressure of arterial carbon dioxide (mmHg); paO2 = partial pressure of arterial oxygen (mmHg); HCO3 = concentration of bicarbonate within arterial blood sample (mMol/L); PEEP = positive end expiratory pressure (mmHg); bpm = breathes per minute; FiO2 = fraction of inspired oxygen; AC/VC = assist-control/volume-control; PS = pressure support; NC = nasal cannula; LPM = Liters per minute ^1^First day of intubation ^2^Ventilator changes made following corresponding ABG values. Repeat ABGs were not obtained because pulse oximeter readings revealed stable oxygen saturations following adjustments. ^3^First day proning was initiated to improve oxygenation & ventilation. Proning occurred in 16-hour intervals.

**Table 3:** Broncheoalveolar Lavage (BAL) Samples Collected on Day 5 of Hospital Course

	RBC	Nucleated Cells	Cell Differentials
BAL #1	28000	388	- Segs: 86%
			- Lymphs: 1%
			- Mono/Mcrphg: 8%
			- Var Lymph: 1%
			- Other: 4%

BAL #2	71000	406	- Segs: 72%
			- Bands: 3%
			- Lymphs: 15%
			- Mono/Mcrphg: 1%
			- Eosins: 1%
			- Other: 4%

BAL #3	76000	511	- Segs: 70%
			- Lymphs: 2%
			- Mono/Mcrphg: 23%
			- Other: 5%

Abbreviations: BAL = Bronchoalveolar Lavage; RBC = Red Blood Cells; Segs = Segmented Cells; Lymphs = Lymphocytes; Mono/Mcrphg = Monocytes/Macrophages; Var Lymph = Various Lymphocytes; Eosins = Eosinophils Of Note: Trend of RBCs on sequential BALs led to the diagnosis of diffuse alveolar hemorrhage (DAH).

**Table 4:** Various Lab Studies Performed to Rule Out Infectious Etiology

Infectious Agent	Type of Test	Source	Results
HIV 1,2 Ag/Ab	Polymerase Chain Reaction (PCR)	Serum	Non-reactive
Respiratory Viral Panel (RVP)^1^	Polymerase Chain Reaction (PCR)	Nasal Swab	Not Detected
Legionella Antigen	Enzyme Immunoassay (EIA)	Urine	Negative
Q Fever Ab IgM/IgG (Phase I-Ii)	Indirect Immunofluorescence Antibody (IFA)	Serum	<1:16
Coccidiodes Antibody	Immunodiffusion (ID) & Complement Fixation (CF)	Serum	Negative
Cocci Immunodiffusion IgM/IgG	Immunodiffusion (ID)	Serum	Negative
Quantiferon TB Gold	Interferon-Gamma (IFN-γ) Release Assay (IGFRA)	Serum	Negative
Hepatitis Panel^2^	Polymerase Chain Reaction (PCR)	Serum	Non-reactive
Acid Fast	Culture	Sputum	No Growth Detected
Gram Stain	Culture	Blood & BAL^3^	No Growth Detected
Viral	Culture	BAL	No Growth Detected
Respiratory	Culture	Sputum	No Growth Detected
Fungal Stain	Culture	Blood & BAL^3^	No Growth Detected

Abbreviations: HIV = Human Immunodeficiency Virus; Ag = Antigen; Ab = Antibody; IgM = Immunoglobulin M; IgG = Immunoglobulin G; TB = Tuberculosis; BAL = Bronchoalveolar Lavage ^1^RVP consists of the following viral PCR test(s): Adenovirus, Metapneumovirus, Rhinovirus/Enterovirus, Influenza A&B, Parainfluenza 1-4, RSV, Bordetella parapertusis, RP, Bordetella pertussis, RP, Chlamydophila pneumoniae, Mycoplasma pneumoniae, RP, Coronavirus HKV1/NL63/229E/OC43 ^2^Hepatitis panel consists of the following antibody (AB) and antigen (Ag) test(s): HAV AB IgM, HBC AB IgM, HBS AB, HBS Ag, & HCV AB *^3^Bronchoalveolar Lavage (BAL) samples as listed above under Table 2*

**Table 5:** Studies Performed to Rule Out Autoimmune Cause

Autoimmunity Tested	Type of Test	Source	Results
C-ANCA/P-ANCA AB^1^	Enzyme Immunoassay (EIA)	Serum	Negative
Antinuclear Antibody (ANA) Panel^2^	Indirect Immunofluorescence Antibody (IFA)	Serum	Negative
Angiotensin Converting Enzyme	Enzyme-Linked Immunosorbent Assay (ELISA)	Serum	20 U/L (ref. range: 16-85 U/L)
C1 Esterase Inhibitor	Enzyme Immunoassay (EIA)	Serum	60 mg/dL (ref. range 19 - 37 mg/dL
C1 Esterase Inhibitor, Functional	Enzyme Immunoassay (EIA)	Serum	> 90 (ref. range: > 67 (Normal) 41-67 (Equivocal) <41 (Abnormal))
C2 Complement	Immunodiffusion (ID)	Serum	63 u/mL (ref. range: 25 - 47 u/mL)
C5 Complement	Immunodiffusion (ID)	Serum	34 mg/dL (ref. range: 10.6 - 26.3 mg/dL)

Abbreviations: AB = Antibody ^1^Antineutrophil Cytoplasmic Antibody (ANCA) testing includes: Anti-myeloperoxidase (MPO) antibodies & anti-proteinase 3 (PR3) antibodies ^2^ANA panel consists of the following antibody (AB) tests: ANA AB Quant, anti-CCP3 AB, anti-Centromere AB, anti-Chromatin AB, anti-RNP AB, anti-SCL-70 AB, anti-Sm AB, anti-SS-A AB, and anti-SS-B AB

## References

[1] Blanco J, Gaines S, Arshad J, Sheele JM (2018). Silicone pneumonitis, diffuse alveolar hemorrhage and acute respiratory distress syndrome from gluteal silicone injections. Am J Emerg Med.

[2] Ng BH, Mat WRW, Abeed NNN, Hamid MFA, Yu-Lin AB, Soo CI (2020). Silicone pneumonitis after gluteal filler: a case report and literature review. Respirol Case Rep.

[3] Schmid A, Tzur A, Leshko L, Krieger BP (2005). Silicone embolism syndrome: a case report, review of the literature, and comparison with fat embolism syndrome. Chest.

[4] Zamora AC, Collard HR, Barrera L, Mendoza F, Webb WR, Carrillo G (2009). Silicone injection causing acute pneumonitis: a case series. Lung.

[5] Chastre J, Basset F, Viau F (1983). Acute pneumonitis after subcutaneous injections of silicone in transsexual men. N Engl J Med.

[6] Seitz R, Schramm W (2020). DIC in COVID-19: Implications for prognosis and treatment?. J Thromb Haemost.

[7] (2019). Aesthetic Plastic Surgery National Databank Statistics 2019. [online] Available at.

[8] Scudder C (2020). Dallas woman faces murder charge after police say she gave illegal but injection that killed Fort Worth woman.

[9] Adegunsoye AO, Matchet S, Valentino D J (2012). A 20-year-old woman with rapidly progressive dyspnea and diffuse pulmonary infiltrates. Respir Med Case Rep.

[10] Wolf LE, Lappé M, Peterson RD, Ezrailson EG (1993). Human immune response to polydimethylsiloxane (silicone): screening studies in a breast implant population. FASEBJ.

[11] Connors JM, Levy JH (2020). Thromboinflammation and the hypercoagulability of COVID-19. J Thromb Haemost.

[12] Kaufman AE, Naidu S, Ramachandran S, Kaufman DS, Fayad ZA, Mani V (2020). Review of radiographic findings in COVID-19. World J Radiol.

